# Short- and Long-Term Effects of Unpredictable Repeated Negative Stimuli on Japanese Quail's Fear of Humans

**DOI:** 10.1371/journal.pone.0093259

**Published:** 2014-03-25

**Authors:** Agathe Laurence, Sophie Lumineau, Ludovic Calandreau, Cécile Arnould, Christine Leterrier, Alain Boissy, Cécilia Houdelier

**Affiliations:** 1 Ethos, UMR 6552- Université de Rennes 1- CNRS, Rennes, France; 2 UMR 85 Physiologie de la Reproduction et des Comportements, INRA, Nouzilly, France, UMR 6175, CNRS, Nouzilly, France, Université de Tours, Tours, France, IFCE, Nouzilly, France; 3 INRA UMR 1213, Unité de Recherche sur les Herbivores, INRA Vet-Agro Sup, Saint-Genès Champanelle, France; Institut Pluridisciplinaire Hubert Curien, France

## Abstract

Numerous aversive events occur in poultry production, and if repeated and unpredictable, can result in an impaired welfare. Some events such as handling can be perceived negatively and it is of interest to understand how humans' behaviour could affect poultry's behaviours and especially its avoidance of humans. Our aim was to evaluate short- and long-lasting effects of a 3-week procedure involving unpredictable repeated negative stimuli (URNS) applied during the post-juvenile period on quail's reactivity to humans. We compared the reactions of two sets of quail: URNS was applied to one set (treated quail) and the other set was left undisturbed (control quail). When two weeks old, treated quail were exposed to a variety of negative stimuli, either applied automatically or involving human presence. One and seven weeks after the termination of the procedure, the reactivity of control and treated quail to a passive human being was evaluated. Furthermore, the experimenter with her hand on a trough containing a mealworm assessed the propensity of quail of both groups to habituate to feed close to a human being. In the presence of a seated observer, treated quail were more inhibited and more alert than control quail. Likewise, seven weeks after the end of the URNS procedure, more treated than control quail adopted a fear posture. Moreover, whereas control quail spent as much time in the different areas of their cages, treated quail spent more time in the rear part of their cages. Finally, whereas control quail habituated gradually to feed near the experimenter's hand, treated quail did not. All these tests evidence negative short- and long-term effects on treated quail's reactivity to a passive human being and on their habituation to a human being when her presence is positively reinforced. This highlights the importance of young poultry's experience with humans in production.

## Introduction

In animal production, numerous repeated events (e.g. crating, sudden noises or unknown conspecifics) could result in chronic stress, thus impairing welfare. Chronic psychological stress is the result of repeated unpredictable aversive events [1]. Chronic stress procedures, based on that definition, were first applied to rodents to produce depression-like symptoms and thus create a model of human psychological disorders (for a review, see [2], [3]). These studies reported numerous effects of chronic stress procedures on rodents' physiology (e.g. corticosterone concentration, body weight) and behaviour (e.g. sleeping patterns, aggressiveness, spatial learning). However, chronic stress effects on other animal models such as birds and fish have been investigated only recently [4]–[6]. To our knowledge, birds' behavioural modification due to unpredictable repeated negative stimuli exposure has only been studied in Japanese quail (*Coturnix Coturnix japonica*). In these studies, authors reported increased fearfulness in novel environments [7] and behavioural modifications of activity in their home cage (e.g. increased resting rate) after two weeks of chronic psychological stress using unpredictable and repeated negative events [8].

Negative events encountered under production conditions (e.g. rough handling, vaccination) can be associated directly to stockpeople or to their presence, whereas positive events such as automatic food distribution could not [9]. In addition to increased fear reactions in the presence of human beings, strong fear of humans has been correlated with detrimental effects on production such as food conversion efficiency in egg- and meat-type stock birds [10]–[13]. Thus, numerous studies have investigated how humans' behaviour could affect poultry's and especially domestic chicks' (*Gallus gallus domesticus*) avoidance of human. Most studies focused on the effects of handling at different developmental stages and reported that handling young chicks regularly, either gently (picked up and stroked) or roughly (suspended by the legs) decreased their avoidance of an experimenter (e.g. [14], [15]). Handling, but not rough handling, decreased tonic immobility (a catatonic state in response to fear stimuli which can be artificially induced by light manual restraint) durations, a common indicator of general fearfulness in poultry [14], [16]. Furthermore, increased visual contact with an experimenter alone was sufficient to decrease avoidance of humans and/or tonic immobility durations [14], [16]. However, other studies reported that the qualitative aspect of visual contact between human beings and poultry such as speed of movement were positively correlated with poultry's avoidance of humans and with the amount of their fear reactions [17], [18].

Because poultry are exposed to numerous negative events potentially implying humans, this could have an impact on the development of human-poultry interactions. In this context, studying how repeated exposure to negative stimuli and reactivity to humans interact is fundamental in welfare research, as negative relationships with humans pose ethic problems in terms of fear reactions.

The first aim of this study was to investigate the effects of unpredictable repeated negative stimuli on Japanese quail's fear of humans. This procedure is applied during the post-juvenile stage and consists in the application of various events inducing negative emotions (e.g. as physical restraint, loud noises or food delay). Our second aim was to test whereas behavioural modifications in treated quail could influence the establishment of a later positive human-quail relationship. Considering the fear of humans, previous studies reported positive effects of increased neutral (standing motionless), positive (stroking a conspecific) or negative (suspending a conspecific by the legs) predicted and repeated visual contact between experimenters and poultry [14]–[17]. By contrast, our procedure involved unpredictable negative events, and some of them could be associated with the presence of humans. Hence, we assessed the responses to humans of quail after they had been exposed 1) to a unpredictable repeated negative stimuli (URNS) procedure (treated quail) or 2) only to regular human presence (control quail). We predicted that treated quail would be more fearful in the presence of the experimenter and that the establishment of a positive relationship with her would require more trials than for control quail. Two test situations assessed short-term effects: a motionless human in the room and repeated presentation of appetizing food near a human's hand, inside the cage. This test aimed to evaluate whether our procedure had influenced the possibility to establish a positive human-quail relationship by presenting appetizing food [19], [20]. In order to investigate the strength of our URNS procedure effects on behaviours, we also tested long- term effects on human-quail interactions through the quail's responses to a standing human in the rearing room. Thus, we could evaluate whether several weeks after the termination of URNS procedure, behavioural differences between control and treated quail would remain or not.

## Methods

### 1. Ethics statement

Animal care procedures were conducted in accordance with the guidelines set by the European Communities Council Directive (86/609/EEC) and French legislation on animal research. Our protocol was approved by the regional ethic committee (CREEA: “*Comité Rennais d*'*Ethique en Expérimentation Animale”* meaning “Rennes city's Ethics Committee for Animal Experimentation”: agreement n°R-2011-SLU-01).

### 2. Animals and housing conditions

Our subjects were 43 female Japanese quail (*Coturnix coturnix japonica*). Eggs issued from a controlled genetic line [21] were provided by the experimental unit 1295 (UE PEAT) and UMR 85, Physiologie de la Reproduction et des Comportements, INRA Centre Val de Loire, Nouzilly, France. They were incubated for 17 days in a collective incubator in our laboratory (38.5°C, 45% of humidity for 15 days and 65% of humidity for the last two days). After hatching, quail were reared in groups (males and females) of 30 chicks in cages with litter (93×45×32 cm). During the 2 weeks following hatching, cages were provided two heating lamps (38±1°C) and a green light (30 lux) dimly lit the rooms during the night to help the chicks locate the heat source. After this period, the chicks were able to regulate their body temperature and the ambient temperature was maintained at 19±2°C. When they were 17 days old, their sex was determined relying on feather dimorphism, and males and females were then reared separately (males were included in another study). They were wing-tagged and transferred to individual battery cages (24.5×35×20 cm). A 12:12 h light-dark cycle was maintained during all the experiment (lights on at 9:00 am). Water and food (cereal diet in the form of mixed pellets for chicks and granulates for adults: crude protein = 21%; lysine = 1.26%) were provided *ad libitum*. When they were 11.5 weeks old, females were transferred to individual cages (51×40×35 cm) for further testing.

### 3. Unpredictable repeated negative stimuli URNS procedure

URNS began when the quail were transferred to the batteries (post-hatch day 17) and lasted for 21 days (until post-hatch day 38). It was applied to half of the subjects (two rooms, treated group, N = 22) whereas the other half (the other two rooms, control group, N = 21) was left undisturbed although the experimenter visited them regularly. The URNS procedure consisted of ten negative stimuli applied randomly, during both night (one stimulus) and day (three to five different stimuli), with variable durations (from 2 to 180 min). This reduced the possibility of habituation to the negative stimuli, as unpredictability is known to enhance stress reactions in domestic fowl [22]. The stimuli used have been tested and described in previous studies [7], [8], [23]. Briefly, four stimuli were applied individually: metal stick on cage rods twice in 2 min (noise and suddenness), air or water spurted twice in 30 min, physical restraint in a cage corner for 30, 40 or 50 min. The five other stimuli were applied to the entire battery in the same time: ventilators starting automatically and repeatedly for 5 to 15 min at night; food accessibility delayed by placing transparent devices on the troughs for 3 hours just before daytime; unexpected sounds (100 dB) lasting 4 seconds three times in 2 to 10 min, night and/or day. This stimuli was composed of three different sounds, none having any biological signification for quail, and the call of a hawk (natural predator). Finally, all quail from a same battery were put together in a transport cage (78×43×13 cm). This transport cage was placed on a cart and rolled about in the facility for 20 minutes. We also applied two social perturbations: quail were moved to another cage in the same battery in order to change their neighbours. This procedure was applied twice during the URNS procedure (on days 5 and 9). In addition, for one hour, a quail was put in the home cage of another quail, within the same battery. Thus, two non-familiar quail were put together in one individual cage of the battery. This social perturbation was applied twice during the procedure (on days 8 and 12), so that each quail was once the resident, and once the intruder.

All interventions (care, stimuli, measurements and tests) involved the same experimenter. To differentiate the human being intervening in different situations, she was dressed in white (lab coat) and blue (mask, gloves, and shoes) when stimuli were applied, otherwise she was dressed in green (overall) (i.e. observations and care giving).

The percentage of time stimuli implying no human presence were applied during the entire procedure was approximately 40% (e.g. ventilators), that involving human visual contacts was approximately 10% (e.g. metal stick on the cage rods) and that involving handling was approximately 50% (e.g. social perturbations).

### 4. Measurements

As indicators of the quail's development, growth (i.e. weight difference between two weeks) was measured during and after the URNS procedure, until quail were 8.5 weeks old, yielding five measures. In addition, when juvenile moult began (around 5 weeks after hatching), we assessed for each quail weather it has started, by checking the presence or absence of growing feathers (on the head or neck and/or on the rest of the body) [24]. This was evaluated four times, when quail were 5 weeks old (end of the URNS procedure), and when they were 6, 7 and 8.5 weeks old.

### 5. Activity budget

Behavioural modifications were assessed at the end of each week of the URNS procedure on all quail between 8:30 and 12:30. Activity budgets were evaluated using instantaneous scan sampling (32 scans per quail, every 8 minutes). Observation order was randomized between groups in order to avoid any time bias. Moreover, to avoid any effect of human presence, each experimental room was equipped with video cameras before the experiment so that the experimenter could observe the quail on a monitor in another room at any time. The following behaviours were noted: resting (e.g. low or lying posture, eyes closed), observation (e.g. medium posture), preening (stretching, scratching body parts, feather smoothing) and feeding and foraging in the trough (in front of the cage).

### 6. Behavioural tests

#### 6.1. Reactivity to a motionless observer: short-term effects on time-budget

One week after the end of the URNS procedure (URNS+1), the experimenter, dressed in green (all quail saw the experimenter in that outfit for the same amount of time during the whole experiment), sat 1.50 m in front of the battery. Then, the quail's behaviour was monitored by scan sampling for 1 hour in each room alternatively, between 8:30 and 12:30. Behaviours were noted every 2 minutes, yielding 30 scan data for each subject. The behavioural items as above were recorded (see 2.5. Activity budget) and also vigilance (head and body stretched) and fear posture (low posture with head near to the ground and below the body) which could be displayed in reaction to frightening stimuli.

#### 6.2. Reactivity to a motionless observer: long-term effects on time budget

Seven weeks after the end of the URNS procedure (URNS+7), all females were moved to larger individual cages in the same room (no visual contact between females). Inside the room, the position of control and treated quail's cages was balanced between rows and lines. The standing experimenter dressed in green scanned the cages, moving between each row of cages (17 rows of 2 or 3 cages). Behaviours were noted for 6 hours, every 8 minutes, yielding 40 scan data for each quail, between 8:30 and 14:30. The same behavioural items as above were recorded. As these cages were larger than the battery cages, the position of the quail (head and feet) was noted in addition (rear, middle, and front of the cage).

#### 6.3. Habituation to the experimenter presenting a mealworm

After the end of the URNS procedure, all quail were given mealworms in a small opaque trough placed inside their cage, for three consecutive days to habituate them to this food type. Mealworms are highly appetizing food for quail and all quail used in this test ate the worms, each day of the habituation phase.

Two weeks after the end of the URNS procedure (URNS+2), the same trough containing mealworms was placed in the front part of the cage. Through the half-open cage door, the experimenter placed her hand on half of the trough, without preventing the quail from eating the mealworms. Then, the latency to come to the trough containing the mealworms was noted. This test was replicated four times with the opaque trough (one test per day) and once, a final test with a transparent trough, so that the possibility to see the mealworms could increase the quail's motivation to approach the trough. When a test quail did not come to eat the mealworms, the test was ended after 2 minutes. These tests took place between 14:00 and 17:00 and the testing order was balanced between groups each day so that there was no time bias.

### 7. Statistical analysis

Normality and homoscedasticity of the residuals were verified for growth and activity budget data. Frequency for each behaviour (number of occurrence of a given behaviour/total number of scans) were extracted from scan samplings (activity budget and reaction to the experimenter). Growth and activity budget behaviours were analysed using mixed models (URNS × time) and with individuals as a random factor. Feeding data were arcsine root transformed to achieve a normal distribution of the residuals. Interactions between weeks and URNS were analysed with post-hoc Tukey HSD tests. Because of minor health problems (diarrhea), body weight data were missing for two control quail which were thus removed for growth analyses.

Other behavioural data residuals (short and long-term effects on time budgets, habituation to the experimenter presenting a mealworm) did not fit a normal distribution, thus non-parametric statistics were used. For each parameter, Mann-Whitney U-tests compared treated and control quail. When a test was repeated, data were compared with Friedman tests conducted within each group. Data are presented as means ± standard error of the mean (SEM). Moreover, the numbers of quail that displayed and that did not display a given behaviour (e.g. adopting a fear posture or approaching the experimenter) were compared with G–tests, and with a Fisher test when samples were small. These data are presented as the number of quail that expressed the behaviour divided by the total number of quail for each group, in percent. All tests were performed using Statistica (Statsoft, Inc.) and R softwares. The significance level was set at P≤0.05 (two-tailed) and tendencies were considered up to P<0.10.

## Results

### 1. Growth and moulting

**Figure 1:**
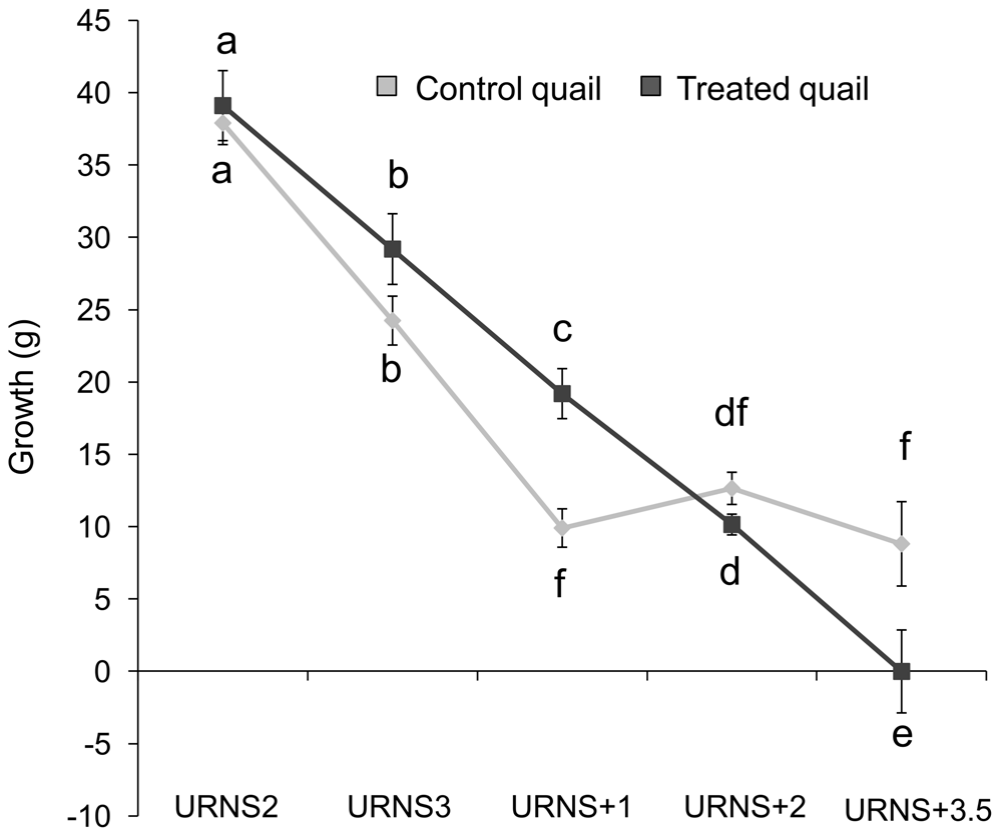
Body weight gain between weeks (mean in mm ± SEM). URNSX: increase between X-1 and X week(s) after the end of the URNS procedure. Control quail: N = 19, treated quail: N = 22. Post-hoc Tukey HSD tests: different letters means significant differences.

Growth was significantly influenced by time but not by the URNS procedure (time: F_(4,156)_ = 85.7, P<0.0001; URNS: F_(1,156)_ = 0.2, P = 0.67, fig. 1). In addition, there was a significant interaction between the URNS procedure and time (F_(4,156)_ = 5.5, P = 0.0003). Post-hoc comparisons evidenced that one week after the end of the URNS procedure (URNS +1), growth stopped decreasing in control quail (Tukey HSD tests between weeks: URNS2/URNS3: P<0.0001; URNS3/URNS+1: P<0.0001; URNS+1/URNS+2: P = 0.99; URNS+2/URNS+3.5: P = 0.96; fig. 1) whereas it kept decreasing until URNS+3.5 in treated quail (URNS2/URNS3: P = 0.02; URNS3/URNS+1: P = 0.02; URNS+1/URNS+2: P = 0.05; URNS+2/URNS+3.5: P = 0.01; fig. 1). In addition, treated quail gained significantly more weight than control quail at URNS+1 (Tukey HSD tests between groups: P = 0.05) and control tended to gain more weight at URNS+3.5 (P = 0.08).

**Figure 2:**
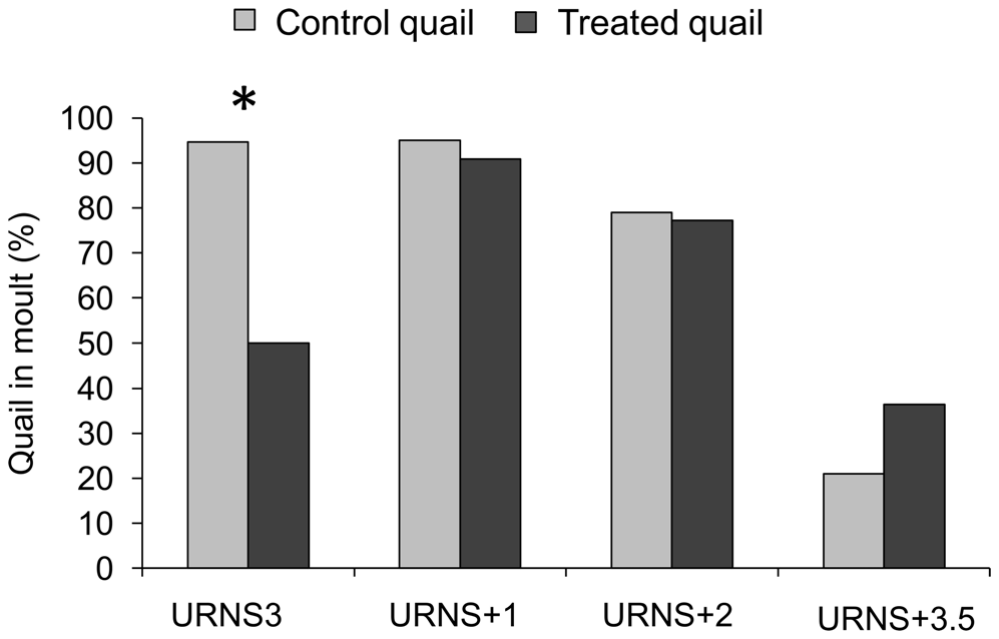
Percent of quail in moult in each group in relation to time. URNS3: end of procedure, URNS+X: X week(s) after the end of the procedure. * P<0.001 (Fisher exact test). Control quail: N = 19, treated quail: N = 22.

In parallel, just after the URNS procedure ended (URNS+0), whereas almost all control quail had started their moult, only half of the treated quail had and this difference was significant (Fisher exact test, P = 0.002, fig. 2). After that, at URNS+1, URNS+2 and URNS+3.5, a majority of treated quail also began their moult and no differences between the two sets were evidenced (Fisher exact tests, P>0.05).

### 2. Activity budget (Table 1)

Globally, preening and observation frequencies increased when quail grew up, whereas resting and eating frequencies decreased.

**Table 1:** Control quail's (N = 21) and treated quail's (N = 22) activity budgets (mean rates ± SEM) during URNS procedure.

		Control	Treated	URNS effect	Time effect	URNS x time
	week 1	25.3±1.9	18.6±1.7			
**Preening**	week 2	30.5±2.8	25.7±2.2	F_(1,82)_ = 5.34 *	F_(1,82)_ = 21.36 *	F_(2,82)_ = 0.36
	week 3	36.3±1.6	31.5±2.9			
	week 1	13.7±1.7	15.6±2.1			
**Observation**	week 2	11.5±1.7	16.1±2.1	F_(1,82)_ = 3.34 ^#^	F_(1,82)_ = 8.92 *	F_(2,82)_ = 0.36
	week 3	17.7±1.8	22.7±2.5			
	week 1	28.2±3.1^a^	36.8±2.2^b^			
**Resting**	week 2	33.2±3.0^ab^	35.2±3.0^ab^	F_(1,82)_ = 0.25	F_(1,82)_ = 4.04 *	F_(2,82)_ = 4.25 *
	week 3	30.2±3.0^abc^	23.7±3.1^c^			
	week 1	16.2±1.6	14.8±2.0			
**Feeding**	week 2	9.2±1.8	12.2±1.7	F_(1,82)_ = 0.58	F_(1,82)_ = 11.04 *	F_(2,82)_ = 1.16
	week 3	6.8±1.3	9.1±2.5			

Linear mixed model, *P<0.05, #P<0.08.

During the 3 URNS weeks, treated quail preened significantly less than did control quail and they tended to observe their environment more often. Finally, there was a significant interaction between URNS and time for resting frequencies. Post-hoc LSD Fisher tests revealed that treated quail rested more than control quail at the end of the first URNS week (P = 0.038). Moreover, treated quail (P = 0.003) but not control quail (P = 0.60) rested significantly less after three weeks of URNS. No significant effects on feeding could be evidenced (URNS: P = 0.39; URNS x week: P = 0.35).

### 3. Reactivity to a motionless observer

#### 3.1. Short-term effects

**Figure 3:**
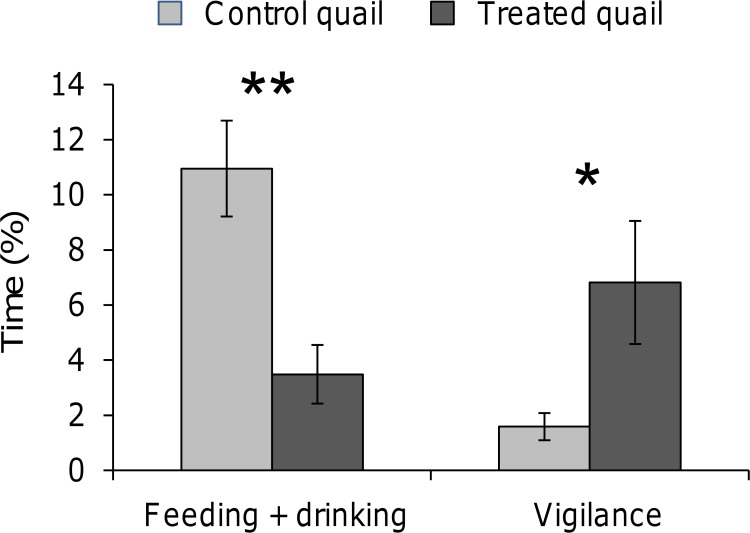
Feeding and drinking and being vigilant (% time, mean ± sem) for control and treated quail in the presence of the observer. Mann-Whitney U-tests, **P<0.01, *P<0.05. Control quail: N = 21, treated quail: N = 22.

Quail's reactions in their home-cage to the static presence of an observer in the room differed significantly between treated and control quail. First, treated quail spent significantly less time feeding and drinking (Mann-Whitney U-test, U = 104.0, P = 0.002, fig. 3) whereas they spent significantly more time being vigilant (U = 145.0, P = 0.025, fig. 3). Second, in the presence of the observer, no quail from the treated group were observed in a lying posture, whereas 19% of the control quail were observed in this posture (Fisher exact test, P = 0.049). Other behaviours (fear postures, resting, observation and preening) did not differ between control and treated quail (P>0.05).

#### 3.2. Long-term effects

**Figure 4:**
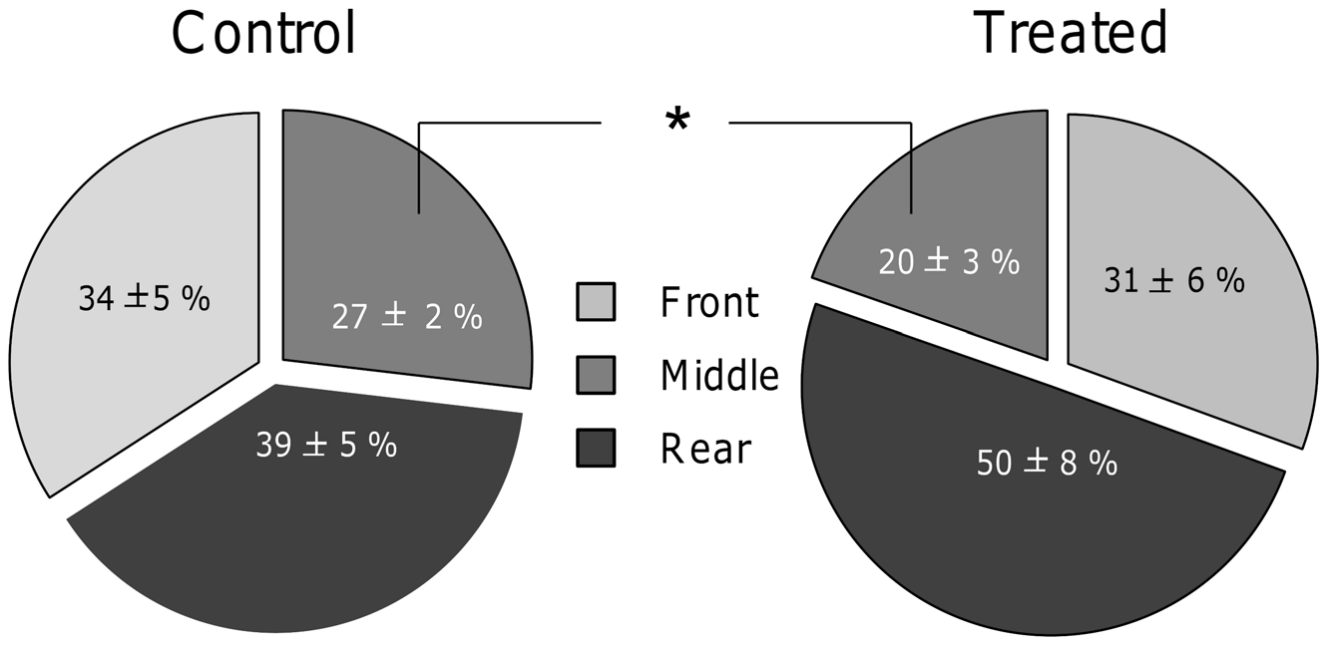
Spatial distribution in home-cage (% time in different cage zones – front, middle, rear, mean ± SEM) of control and treated quail, when an observer was present, seven weeks after the URNS procedure. Chi square tests compared the relative times spent in the three zones to chance for each group and Mann-Whitney U-tests compared proportions of time spent in each zone between control and treated quail: *P<0.05. Control quail: N = 21, treated quail: N = 22.

Seven weeks after the end of the URNS procedure, the reactions of control and treated quail in the presence of an observer still differed. First, significantly more treated than control quail adopted a fear posture (observation in low posture) when the observer was present (treated quail: 27%, control quail: 5%, G-test, G = 4.4, df = 1, P = 0.036). In addition, whereas control quail remained approximately as long in the three cage zones of their cage (front, middle, rear) (Chi square test, χ^2^ = 1.1, df = 1, P = 0.57), treated quail spent more time in the rear (χ^2^ = 6.7, df = 1, P = 0.03) and significantly less time in the middle of their cage than did control quail (Mann-Whitney U test, U = 146.5, P = 0.04, fig. 4). Finally, resting, observation and preening and feeding did not differ between the two groups of quail (P>0.05), seven weeks after the URNS procedure.

### 4. Habituation to an experimenter presenting a mealworm

**Figure 5:**
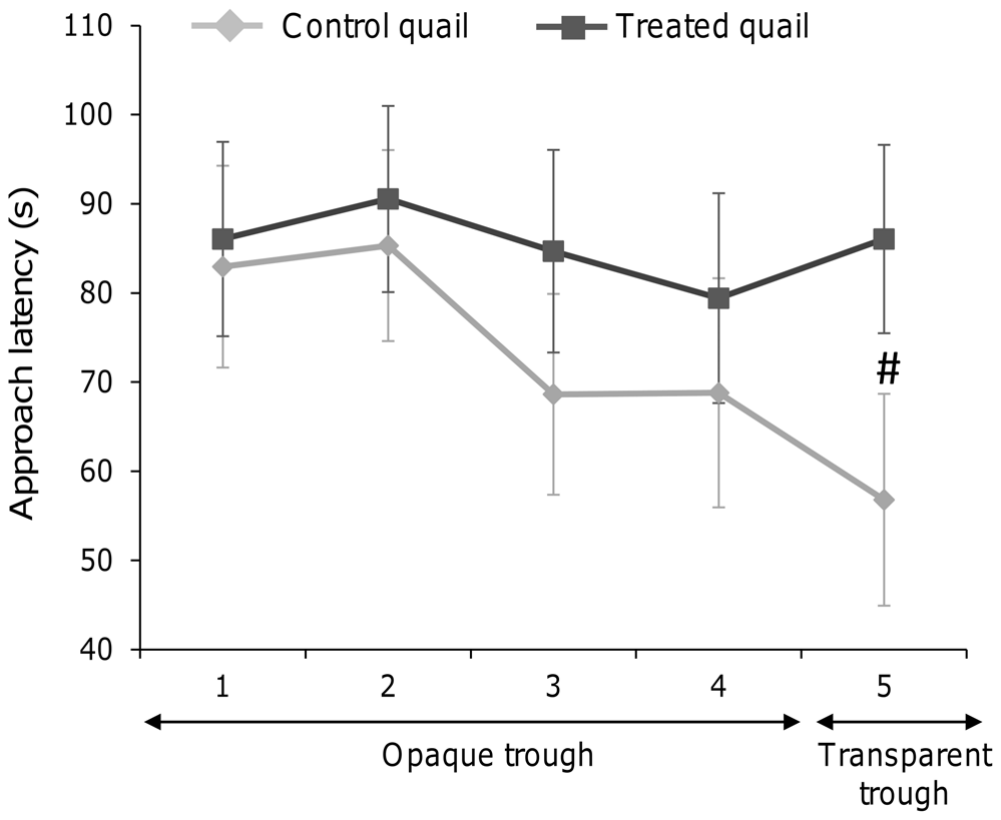
Approach latencies of the trough containing a mealworm (mean ± SEM, in seconds), with the experimenter's hand on it, for control (squares) and treated (diamonds) quail during the four trials with an opaque trough and a fifth trial with a transparent trough. Mann-Whitney U-tests compared control and treated quail: # P<0.10). Control quail: N = 19, treated quail: N = 22.

Two weeks after the end of the URNS procedure, approach to a mealworm close to the experimenter's hand differed between control and treated quail and it depended on the device used. When a mealworm was presented in a trough on which the experimenter had laid her hand, approach latencies of control quail but not of treated quail decreased progressively (Friedman test, control quail: χ^2^ = 10.7, df = 4, P = 0.03; treated quail: χ^2^ = 6.5, df = 4, P = 0.17, fig.5). During the last trial, control quail tended to approach the trough faster than did treated quail (fig. 5). Finally, significantly fewer treated quail approached the mealworm placed in a transparent trough than did control quail (control quail: 68%, treated quail: 36%, G-test: G = 4.3, df = 1, P = 0.039).

## Discussion

Here, we demonstrated first that our URNS procedure influenced developmental parameters in Japanese quail, and that their behaviour is altered even in the absence of any stimulus. Second, our study shows that a URNS procedure, partly involving human presence, increased fear reactions in the presence of an experimenter, soon and long after the termination of the procedure, and decreased treated quail's ability to habituate to the experimenter offering appetizing food.

In the present study, we showed temporal differences in the growth of control and treated quail. From the beginning to the end of the URNS procedure, growth was similar between control and treated quail. On the contrary, previous chronic psychological stress studies in birds, using repeated exposure to negative stimuli, evidenced weight loss in stressed birds (e.g. European starlings: [4]), as it was reported in original studies in rodents (for a review: [2], [3]). However these studies were conducted in adult subjects, in which body weight should be stable as their growth is over. In the present study we assessed developmental effects of URNS, and it is likely that the fast growth occurring during the juvenile period alleviated URNS effects. Nonetheless, at 5 weeks of age, when growth stabilize in control quail, it kept decreasing in treated quail. During this period, i.e. after the end of the URNS procedure, differences in body weight gain appeared between control and treated quail, the latter gaining the more weight at URNS+1. These differences could be linked to the delay in moult observed in treated quail. Indeed, after the end of the URNS, if control quail gained less weight than treated quail, they also had started their moult earlier, involving the mobilisation of large reserves of energy [25], [26]. After that, growth was similar in both groups, even though 3.5 weeks after the end of the URNS procedure, control quail tended to gained more weight than treated quail which had at this time also started their moult. Hence, our URNS procedure altered the developmental pattern of Japanese quail. This could be linked to brief plasma corticosterone differences between control and treated quail, as this hormone is involved in energy metabolism and has been shown to be influenced by chronic psychological stress procedures [27]. This could be supported by a thorough study of plasma concentrations in the growing feather during the procedure [28].

In addition to developmental data, we reported behavioural differences between control and treated quail during the URNS procedure, when no perturbations (neither negative stimuli nor human presence) were being applied. First, treated quail rested more than control quail after one week of URNS procedure. Sleep is closely linked to energy conservation, and this increase of sleep duration could be a consequence of a disturbed homeostasis [29], [30]. Furthermore, quail were disturbed once per night and the increase of sleep during the light phase could be a way to compensate during the first week of URNS procedure. Finally, preening was disturbed during all the URNS procedure. Concordantly with our present results, a decrease of self care is a common indicator of chronic stress in mammals (e.g. [3]) and this has previously been reported in birds placed on a dust-bathing substrate [8].

One week after the termination of the URNS procedure, treated quail expressed more vigilance in the presence of the experimenter standing motionless. In addition, they fed and drank less than did control quail in the presence of the experimenter but not when she was absent, as shown at the end of the URNS procedure. Previous authors have linked behavioural inhibition to poultry's fear reactions [22]. This author reported that when an individual is frightened, fear competes with other motivational systems, inducing inhibition of feeding, exploration and sexual activity. Our data show that human presence is more frightening for treated than for control quail. We hypothesized first that treated quail's higher reactivity to a passive human being is the result of a general increase of fearfulness, and it is known that poultry species generally perceive human beings as frightening stimuli [31]. In fact, we evidenced previously that the URNS procedure increased emotional reactivity (i.e. the propensity to be easily frightened [22]) [7], [8]. This would be concordant with a study on divergent lines of Japanese quail [32]. In this study, the authors report a positive correlation between general fearfulness, a result of divergent selection on tonic immobility durations, and the capture rank of quail (individuals captured first are considered less frightened by the human experimenter [32]). However, Satterlee and Jones [33] evidenced no differences on the capture rank of two lines of Japanese quail which were selected on their plasma corticosterone concentration in response to restraint, inducing differences in their general fearfulness. Actually, several studies on poultry evidenced that selection on one trait of fearfulness do not systematically implied an increased fearfulness in different frightening situations and thus highlights the multidimensional aspect of fear [34]–[36]. Furthermore, Bertin and Richard-Yris [20] exposed Japanese quail to humans using visual contacts, tactile contacts and offering food. They report a decrease of fear behaviours in the presence of the experimenter, but no effects on general fearfulness, evaluated by their tonic immobility duration.

Thus, another explanation for the higher fear of humans in treated quail could be that fear reactions we report here could be specific to human presence, instead of a general increase of fearfulness. Indeed, this could be a result of an associative learning between the presence of the experimenter and the application of negative stimuli, which have modified quail's reactivity in her presence afterwards. However, when the stimuli were applied, the experimenter dressed differently. Hence, treated quail might have discriminated between the two situations, that is to say when the experimenter was associated with negative stimuli occurrence or when the experimenter was associated with food distribution. However, our results show that treated quail reacted more strongly to the “neutral or positive” human when she was motionless or presenting appetizing food, and this could imply an absence of discrimination. Though, the ability to discriminate has been evidenced in other poultry species. Indeed, previous reports in domestic chicks and laying hens showed that they were able to discriminate between two different human beings and between different types of clothes [37]–[39]. Despite the quail's ability to discriminate between the two experimenter's outfits, a generalization process might also occur. Regardless of the experimenter's appearance, treated quail displayed fear reactions in her presence, even in a neutral or positive context. In fact, previous studies involving positive human-chick interactions showed that the subsequent decrease of fear of humans was generalized to other handlers [38]. So, interactions with one human being, regardless of their nature, seem to be extended to other humans and generate a general representation of human beings. All in all, the present study evidenced a negative effect of our URNS procedure on human-quail short-term interactions.

Seven weeks after the termination of the URNS procedure, negative effects were still evident. First, more treated quail adopted fear postures in the presence of the experimenter than did control quail. Second, in this test the home cage was larger than in the first test (one week after the URNS procedure) and this allowed quail to retreat to the rear of their cage when a moving human being stopped in front of it. Indeed, treated quail were observed more frequently in the rear if their cages whereas control quail were observed as frequently in the rear, as in the middle and as in the front of their cage. Thus, our results show that our URNS procedure had long-lasting effects on quail, lasting at least up to seven weeks after the termination of the procedure. The URNS procedure probably induced stronger negative emotions than did only gentle handling, to which chicks habituate [12], [14]. To our knowledge, long-term effects on human-animal interactions had not been described in birds. By contrast, this phenomenon has already been reported in mammals, as for example in domestic horses [40]. These authors report a long-lasting effect of neonatal forced handling of foals up to one month later, as they avoid human physical contact.

The URNS procedure altered not only the quail's behavioural reactivity toward a passive human being, but also their ability to establish a positive relationship with the experimenter. Human beings are a frightening stimulus for Japanese quail [31]. In fact, during the first trial, latencies to approach the trough were high in both sets and did not differ between control and treated quail. Nonetheless, control quail's latencies to approach the trough decreased but not those of treated quail and during the last trial, more control quail approached the trough. Our URNS procedure is not likely to have induced a decrease of the mealworm appetence in treated quail. Indeed, to avoid neophobia during our test, quail were given mealworms several times before the test, and all except one (from the control group) ate them. Thus, whereas control quail were able to habituate to feed near a passive human being, treated quail were not, or at least not so quickly. Other studies evidenced that the ability to establish a positive human-animal relationship depended on several factors like strain, emotional reactivity, and period of habituation [19], [41], [42]. According to Murphy & Duncan [19], [43], domestic hens' fear of humans cannot be decreased when hens are adults. Yet, Bertin and Richard-Yris [20] were able to induce adult females to approach the experimenter while they were not used to before. A previous experience may explain such a disagreement since our results show that an adult's previous experience with humans could interact with subsequent attempts to establish a positive relationship.

## Conclusion

Previously, URNS procedure was shown to influence Japanese quail's fearfulness in novel environments [7], [8]. Here we report not only increased fear of humans in treated quail, but also decreased ability to establish positive human-animal relationships. This highlights that the nature of the previous interactions with humans is very important for young individuals, all the more so because these negative effects on human-quail relationship could have long-lasting consequences. Indeed, we report for the first time long-lasting effects of repeated exposure to negative stimuli in poultry, several weeks after the procedure ended, even though the procedure began two weeks after hatching.

Moreover, our results bring a valuable contribution to both fundamental and applied poultry research as in a wide majority of tests, including emotional reactivity tests, a human experimenter is involved. These results encourage us to recommend avoiding unpredictable aversive events since these ones might have long term consequences on the relationship with humans.
